# Vitamin D Status in Polish Military Personnel: Associations with Supplementation, Seasonality, and the GC rs2282679 Polymorphism

**DOI:** 10.3390/nu18183041

**Published:** 2026-09-17

**Authors:** Agata Gaździńska, Magdalena Krzyżanowska, Sebastian Spaleniak, Paweł Jagielski

**Affiliations:** 1 Laboratory of Dietetics and Obesity Treatment, Department of Psychophysiological Measurements and Human Factor Research, Military Institute of Aviation Medicine, Krasińskiego 54/56, 01-755 Warsaw, Poland; mkrzyzanowska@wiml.waw.pl; 2 Department of Internal Diseases and Nephrodiabetology, Medical University of Lodz, Żeromskiego 113, 90-549 Lodz, Poland; sebastian19860@op.pl; 3 Department of Nutrition and Drug Research, Faculty of Health Science, Medical College, Jagiellonian University, Skawińska 8, 31-066 Cracow, Poland; paweljan.jagielski@uj.edu.pl

**Keywords:** 25-hydroxyvitamin D, vitamin D deficiency, GC rs2282679, genetic polymorphism, vitamin D supplementation, military personnel

## Abstract

Introduction/Aim of the study: Vitamin D participates in the regulation of calcium and phosphate metabolism and exerts pleiotropic effects that encompass numerous physiological processes. The study aimed to assess vitamin D status in Polish military personnel and to analyze its association with supplementation, the frequency of consumption of selected products that are sources of vitamin D, demographic and anthropometric factors, seasonality, and the *GC* rs2282679 polymorphism. Methods: The study included 331 soldiers aged 21–65 years, 88.2% of whom were men. 25(OH)D levels were determined by electrochemiluminescence, the frequency of product consumption was assessed using the FFQ questionnaire, and GC rs2282679 genotyping was performed by real-time PCR using TaqMan probes. Suboptimal vitamin D status was defined as a concentration of 25(OH)D < 30 ng/mL. Results: The concentration of 25(OH)D < 30 ng/mL was determined in 50.2% of the subjects. Multivariable analysis showed the following independent factors associated with the suboptimal status of vitamin D: blood collection in the spring, less frequent supplementation and the *GG* genotype of the *GC* rs2282679 polymorphism. Age, sex and BMI showed no independent association with this endpoint. The frequency of the consumption of most analyzed products was not significantly associated with 25(OH)D levels. The concentration of 25(OH)D showed a negative correlation with PTH and homocysteine and a weak positive correlation with inorganic phosphorus. The analysis of the classification tree indicated that the GG genotype identified the subgroup with the lowest 25(OH)D concentration. Conclusions: The suboptimal vitamin D status occurred in about half of the examined soldiers. The results indicated the importance of regular supplementation, seasonality and the *GC* rs2282679 polymorphism in the identification of individuals particularly susceptible to suboptimal vitamin D status.

## 1. Introduction

Vitamin D is a group of fat-soluble secosteroid compounds that serve as precursors of biologically active metabolites with hormonal properties [1,2]. The most important forms are vitamin D_2_ (ergocalciferol) and vitamin D_3_ (cholecalciferol). Cholecalciferol is mainly synthesized in the skin from 7-dehydrocholesterol under the influence of ultraviolet B radiation (UVB; 290–315 nm). It is then hydroxylated in the liver to 25-hydroxyvitamin D [25(OH)D] and subsequently in the kidneys to the active hormonal form, 1,25-dihydroxyvitamin D [1,25(OH)_2_D_3_] [3]. Due to its relatively long half-life and because it reflects vitamin D supply from both cutaneous synthesis and diet, serum 25(OH)D concentration is considered the best marker of vitamin D status [3].

However, the definition of vitamin D sufficiency remains under debate. In the present study, vitamin D status was classified according to Polish and Central European recommendations, in which 25(OH)D concentrations < 20 ng/mL are defined as deficiency, 20 to <30 ng/mL as insufficient or suboptimal status, and ≥30 ng/mL as sufficient [4,5]. This threshold was used to define suboptimal vitamin D status (<30 ng/mL) for the purposes of the present analysis. Nevertheless, it should be noted that the 30 ng/mL threshold is not universally accepted as the optimal target concentration for all adults, and the 2024 Endocrine Society guideline no longer endorses a universal target 25(OH)D concentration of 30 ng/mL for generally healthy populations [6].

Vitamin D participates in the regulation of calcium and phosphate metabolism and is essential for maintaining normal bone metabolism [7]. It affects the differentiation and activity of osteoblasts and the process of osteoclastogenesis, thereby supporting normal osteogenesis [8]. Severe vitamin D deficiency leads to rickets in children and osteomalacia in adults, while chronic deficiency was found to be associated with secondary hyperparathyroidism, increased bone turnover, bone mass loss and an increased risk of fractures, including stress fractures [9,10].

Vitamin D status is influenced by numerous environmental, nutritional and individual factors, including exposure to solar radiation, latitude, season, diet, age and body weight [11,12]. Approximately 80% of vitamin D may come from UVB-induced skin synthesis [13]. However, this process may be limited by sunscreen use, cloudiness, air pollution, age and obesity [14,15]. Natural dietary sources of vitamin D are limited and include mainly oily saltwater fish, fish liver oil, egg yolks and selected species of mushrooms [16,17]. Therefore, fortified food and supplementation play an important role [18]. In Poland, vitamin D fortification is mandatory in the case of margarines and fat spreads, whereas the fortification of milk and dairy products is voluntary and depends on the product and the manufacturer. Therefore, dairy products and fat-containing foods may differ substantially in their vitamin D content. In Poland, vitamin D supplementation is recommended from October to April and throughout the year in individuals with insufficient exposure to sunlight [5]. Vitamin D deficiency remains one of the most common nutritional deficiencies worldwide [19], and in Poland it may affect 50–80% of the adult population [5,20,21].

Although several observational studies linked low 25(OH)D concentrations with cardiovascular, metabolic, immune, neuropsychiatric and other non-skeletal outcomes [8,22,23,24,25,26,27,28,29,30], those associations should be interpreted with caution. Low vitamin D status may also reflect lower sunlight exposure, reduced outdoor activity, obesity, chronic illness, lifestyle factors, or poorer general health rather than a direct causal effect of vitamin D deficiency on those conditions.

Genetic determinants are also an important source of interindividual variability in 25(OH)D concentrations [12]. Particular importance is assigned to variants of the *GC* gene, located on chromosome 4q13.3, which encodes vitamin D-binding protein (DBP), the main carrier protein for circulating vitamin D metabolites. The rs2282679 variant is an intronic single-nucleotide polymorphism (SNP) in the *GC* gene. It was defined as a T>G substitution in the assay used in the present study. The G allele was found to be associated with lower total 25(OH)D concentrations and may, therefore, be considered a risk allele for lower vitamin D status [12,31,32]. This association is probably explained by linkage disequilibrium with functional *GC* variants and by differences in DBP concentration, binding affinity or other DBP-related properties. As most circulating 25(OH)D is bound to DBP, variations in the GC gene may influence the transport, distribution, half-life and the measured total concentration of 25(OH)D. Thus, the *GC* rs2282679 polymorphism may affect vitamin D status mainly through its relationship with DBP-dependent transport and the availability of circulating vitamin D metabolites.

Military personnel constitute a particularly relevant group for the assessment of vitamin D status. Despite high levels of physical activity, soldiers may be exposed to the limited cutaneous synthesis of vitamin D because of uniforms, indoor work and specific service-related conditions [33]. At the same time, adequate vitamin D status may be important for musculoskeletal health, regeneration, immunity, psychophysical capacity and operational readiness [34,35,36]. Previous studies in Polish military personnel indicated that insufficient or deficient vitamin D status might be common in this occupational group [33,34,35,36].

Despite the growing number of studies in civilian populations, data on Polish military personnel remain limited. There is also a lack of comprehensive assessments of vitamin D status in this group that simultaneously include supplementation, dietary frequency data, body composition, seasonality and genetic determinants.

The present study aimed to assess 25(OH)D status in Polish military personnel and to analyze its association with the frequency of consumption of selected vitamin D-source foods, vitamin D supplementation, the *GC* rs2282679 polymorphism, seasonality, age, sex and BMI. Moreover, the analysis included associations between 25(OH)D concentration and selected parameters of mineral and metabolic status, including calcium, phosphorus, parathyroid hormone and homocysteine.

## 2. Materials and Methods

### 2.1. Participants

The study included 331 Polish soldiers on active duty (age: 21–65 years; 88.2% of men), serving in the Air Force (52.9%) and the Land Forces (47.1%).

All participants were Polish soldiers of European origin and were stationed in Poland during the study-related sampling period. None of them was deployed overseas at the time of blood sampling.

The participants were recruited from among soldiers covered by the initiatives carried out as part of the National Health Program for 2021–2025. Individuals who gave their informed consent to participate were included in the study.

The final sample size was determined based on the number of program participants meeting the eligibility criteria for whom a complete set of clinical, biochemical, questionnaire and genetic data was available.

The criteria for inclusion in the study were: active military service, age ≥ 18 years, good health status confirmed by the military occupational medicine service and the lack of diagnosed chronic diseases and disorders affecting bone metabolism. The participants used no drugs that could affect vitamin D metabolism.

The exclusion criteria were: the occurrence of chronic diseases (including liver or kidney diseases, endocrine diseases, autoimmune diseases and malabsorption syndromes), diagnosed skeletal diseases (e.g., osteoporosis, osteomalacia or Paget’s disease) and the use of pharmacotherapy that may affect vitamin D metabolism (e.g., glucocorticoids or anticonvulsants).

### 2.2. Assessment of the Nutritional Status

Anthropometric measurements were performed according to standard procedures. Body height was measured using a stadiometer (Holtain, Crymych, UK) to an accuracy of 0.1 cm, and body weight was measured in light clothing after emptying the bladder.

Body mass index (BMI) was calculated as the ratio of body weight (kg) to the square of body height (m^2^). According to the World Health Organization (WHO) criteria, overweight was defined as a BMI of 25.0–29.9 kg/m^2^, while obesity was defined as BMI ≥ 30.0 kg/m^2^ [37].

Body composition was assessed by the Bioelectrical Impedance Analysis (BIA) with the Tanita MC-790MA segment analyzer (Tanita, Tokyo, Japan).

Waist circumference was measured with an unstretchable tape measure halfway between the lower edge of the costal arch and the iliac crest.

### 2.3. Assessment of the Frequency of Consumption of Food Products That Are a Source of Vitamin D and Supplementation

The assessment of the diet and the use of supplements was conducted using the validated Food Frequency Questionnaire (FFQ) for the Polish population [38], modified for the purposes of this study by extending the response category.

The participants determined the frequency of the consumption of individual products over the past 12 months using an 8-point scale: (1) never or almost never, (2) once a quarter or less, (3) once a month or less, (4) several times a month, (5) once a week, (6) several times a week, (7) daily and (8) several times a day. For the purposes of statistical analysis, the variables describing the frequency of consumption were treated as ordinal variables with values from 1 to 8. The questionnaire recorded the frequency of vitamin D supplementation but did not collect information on the exact supplement dose, formulation or brand.

### 2.4. Laboratory Tests

Fasting blood samples were collected in the morning from the antecubital vein by qualified medical personnel. The serum was obtained after centrifugation and was then analyzed in the Laboratory Diagnostics Department of the Military Institute of Aviation Medicine in Warsaw.

The concentration of 25-hydroxyvitamin D [25(OH)D] was determined by electrochemiluminescent immunoassay (ECLIA) using a Vitamin D Total kit on the Cobas e411 analyzer (Roche Diagnostics, Basel, Switzerland). According to the manufacturer’s performance data for the Elecsys Vitamin D total assay on the Cobas e411 analyzer, repeatability coefficients of variation ranged from 2.4% to 7.5%, while intermediate precision coefficients of variation ranged from 2.5% to 8.5%, depending on sample concentration.

Vitamin D status was classified according to the current guidelines for the population of Central Europe as: deficiency (<20 ng/mL), insufficient concentration (20 to <30 ng/mL), sufficient concentration (30 to 50 ng/mL) and high concentration (>50 ng/mL) [5].

Additionally, concentrations of parathyroid hormone (PTH), total calcium, inorganic phosphorus and homocysteine were determined using standard laboratory methods on Roche Diagnostics and Abbott automated diagnostic platforms. Homocysteine concentration was determined by spectrophotometric method with the Cobas 8000 analyzer (Roche Diagnostics, Basel, Switzerland). The respective reference ranges were 15–65 pg/mL for PTH, 8.4–10.2 mg/dL for total calcium and 0.81–1.45 mmol/L for inorganic phosphorus.

### 2.5. GC rs2282679 Genotyping

The rs2282679 (T>G) polymorphism in the *GC* gene was selected based on literature data [12,31].

Genomic DNA was isolated from cheek swabs (Copan FLOQSwabs; Copan Italia S.p.A., Brescia, Italy; distributed by Interpath Services Pty Ltd., Heidelberg West, VIC, Australia) and then extracted using the High Pure PCR Template Preparation Kit (Roche Diagnostics GmbH, Mannheim, Germany). The quantity and quality of DNA were assessed using the NanoPhotometer NP80 spectrophotometer (Implen GmbH, Munich, Germany). The samples were stored at −20 °C until analysis.

Genetic analyses were performed at the Genetic Laboratory, Department of Biomedical Sciences, Józef Piłsudski University of Physical Education in Warsaw, Poland. Genotyping was performed by real-time PCR using the QuantStudio 1 Real-Time PCR System (Applied Biosystems, Thermo Fisher Scientific, Waltham, MA, USA) and the TaqMan SNP Genotyping Assay kit (C_26407519_10; Applied Biosystems, Thermo Fisher Scientific, Waltham, MA, USA). PCR reactions (10 μL) included TaqPath ProAmp Master Mix (Applied Biosystems, Thermo Fisher Scientific, Waltham, MA, USA), TaqMan probe, and genomic DNA.

Thermal cycle conditions included initial denaturation at 95 °C for 5 min, followed by 40 cycles: 95 °C for 15 s and 60 °C for 1 min. Genotypes were determined using QuantStudio Design and Analysis Software v1.5.1.

Genetic analysis was conducted using a recessive model (GG vs. TT + TG) as the primary approach. Moreover, the comparative analysis of the concentration of 25(OH)D between the three genotypic groups (TT, TG and GG) was carried out. The conformity of genotype distribution with the Hardy–Weinberg equilibrium was assessed using the chi-square test.

### 2.6. Study Period and Ethical Approval

The study was conducted in the years 2023–2025. The seasons were classified based on the date of blood collection. Due to the very low number of observations occurring during the winter, the group was not included in the seasonal analysis.

All research procedures were approved by the Bioethics Committee of the Military Institute of Aviation Medicine in Warsaw (Decision No. 01/2018 dated 9 March 2018, and Decision No. 8/2021 dated 11 August 2021) and conducted in accordance with the principles of the 1964 Declaration of Helsinki and its subsequent amendments. Written informed consent to participate in the study was obtained from all the subjects. Data collection and processing were carried out in accordance with the provisions of the General Data Protection Regulation (GDPR).

### 2.7. Statistical Analysis

Descriptive statistics are presented as the mean ± standard deviation (SD) or the median with the interquartile range (IQR), depending on data distribution. The normality of the distribution of variables was assessed with the Shapiro–Wilk test.

Intergroup comparisons for quantitative and ordinal variables were performed using the Mann–Whitney U test or the Kruskal–Wallis test, depending on the number of groups. Categorical variables were analyzed using the chi-square test. Associations between two variables were calculated using Spearman’s rank correlation coefficient.

Multivariable logistic regression was used to identify factors independently associated with suboptimal vitamin D status, defined as 25(OH)D concentrations < 30 ng/mL. The 25(OH)D status < 30 ng/mL (yes/no) constituted a dependent variable. The following covariates were included in the model: age, sex, BMI, season, frequency of vitamin D supplementation and the genotype of the GC rs2282679 polymorphism. The results are presented as odds ratios (OR) with 95% confidence intervals (95% CI). Furthermore, classification trees were used to divide the set of observations into subsets differing in the serum level of vitamin D. Suboptimal vitamin D status, defined as a concentration of 25(OH)D < 30 ng/mL (yes/no), constituted the dependent variable. The following covariates were included in the model: age, sex, BMI, season, frequency of vitamin D supplementation and the genotype of the GC rs2282679 polymorphism. CRT was used as a tree-building algorithm.

The rs2282679 polymorphism was analyzed in the recessive model as the primary approach. Moreover, an exploratory analysis of 25(OH)D concentrations was performed between the three genotypic groups (TT, TG and GG).

All analyses were carried out using PS IMAGO PRO 10 (IBM SPSS Statistics 29, Predictive Solutions, Krakow, Poland; IBM Corp., Armonk, NY, USA). The level of statistical significance was assumed at *p* < 0.05.

## 3. Results

### 3.1. Nutritional Status Assessment

Table 1 shows the characteristics of the study group, which included 331 soldiers (292 men and 39 women) with an average age of 39.4 ± 9.3 years and a mean BMI of 27.5 ± 4.7 kg/m^2^. The median concentration of 25(OH)D was 29.9 ng/mL. Men were older and had higher values of most anthropometric parameters and body composition than women (*p* < 0.05), while women had a higher body fat percentage (%BF) (*p* < 0.001). No significant sex-related differences were observed in the concentration of 25(OH)D, total calcium, phosphate, PTH or homocysteine (*p* > 0.05).

According to the BMI classification, 31.4% of the soldiers had a normal body weight, 44.7% were overweight, and 23.9% were obese (Table 2). Women were more likely than men to have a normal body weight (66.7% vs. 26.7%; *p* < 0.001). The analysis of body fat content showed significant differences between the sexes (*p* < 0.001). Over half of men (57.6%) were classified as overfat or obese. As regards women, the percentage was 25.6%.

**Figure 1:**
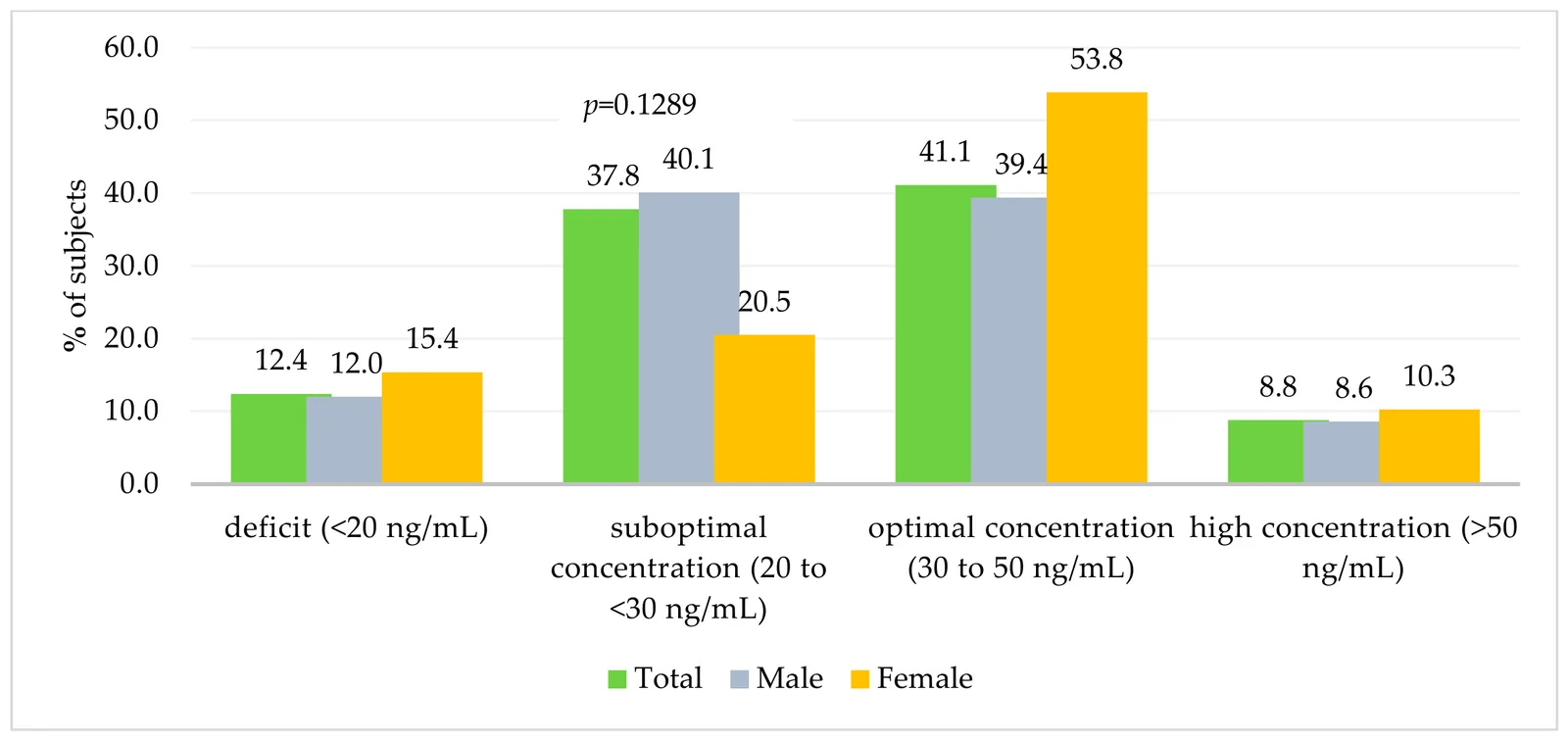
Distribution of serum 25(OH)D concentration categories according to sex among the studied soldiers.

The distribution of 25(OH)D concentrations in the study population is shown in Figure 1. Vitamin D deficiency (<20 ng/mL) was found in 12.4% of the participants, while 37.8% were characterized by suboptimal concentrations (20–29 ng/mL). Sufficient concentrations of 25(OH)D according to the adopted classification (30–50 ng/mL) were observed in 41.1% of the subjects, and high concentrations (>50 ng/mL) in 8.8%. No significant differences between men and women were noted with regard to the distribution of 25(OH)D concentration categories. The percentage of individuals with vitamin D deficiency was comparable in both groups (15.4% in women vs. 12.0% in men).

No significant differences between sexes were noted for the distribution of vitamin D status categories (χ^2^ test, *p* = 0.1289).

The concentration of 25(OH)D differed significantly across BMI categories (*p* = 0.0051; Table 3). Vitamin D concentration tended to decrease with an increase in BMI. The median 25(OH)D concentration was 31.53 ng/mL (IQR: 24.42–41.04) in individuals with normal body weight, 29.97 ng/mL (IQR: 25.00–38.20) in overweight individuals, and 27.32 ng/mL (IQR: 20.00–34.00) in those with obesity.

A similar association was observed in relation to fat content (*p* = 0.004). In participants with normal or reduced body fat, the median concentration of 25(OH)D was 31.96 ng/mL (IQR: 25.00–41.93), while in the group with excessive body fat content corresponding to obesity, it was 27.52 ng/mL (IQR: 20.00–34.86). The results indicated that higher body weight and higher fat content were associated with a lower concentration of 25(OH)D in the studied population of soldiers.

As BMI may overestimate adiposity in physically active individuals with high muscle mass, BMI results should be interpreted with caution in military populations. Therefore, BMI was considered together with body fat percentage in the present analysis. The parallel decrease in 25(OH)D concentrations across both BMI categories and body fat categories suggests that, in this study group, BMI reflected adiposity to a considerable extent, although it cannot fully replace direct body composition assessment.

### 3.2. Frequency of the Consumption of Selected Food Products That Are Dietary Sources of Vitamin D

The frequency of the consumption of products that are sources of vitamin D is shown in Table 4. Fatty and lean fish were most commonly eaten several times a month or less frequently (approx. 60% in total). Conversely, eggs were often present in the diet, i.e., 53.5% of the respondents consumed them several times a week, and 12.4%—daily.

Among fat-containing products, vegetable oils were most frequently consumed at least several times a week (58.2%). Butter was also commonly consumed (61.9% at least several times a week), whereas margarine was consumed rarely (63.8%—never or sporadically). Dairy products constituted an important part of the diet—66.9% of the respondents consumed milk and natural milk beverages at least several times a week, and cheeses and curd cheeses were regularly consumed by over 40% of the participants.

In the sex-stratified analysis, significant associations were observed in men for the consumption of eggs (ρ = 0.167; *p* = 0.004) and omega-3 acid supplements (ρ = 0.149; *p* = 0.011). In women, the associations were significant only for the consumption of sweetened milk drinks (ρ = −0.381; *p* = 0.017). The remaining correlations revealed no statistical significance. Spearman’s correlations between the frequency of the consumption of selected foods that are sources of vitamin D and the concentration of vitamin D depending on sex are presented in the Supplementary Table S1.

### 3.3. Frequency of Supplementation and Total Vitamin D Levels in the Blood of Soldiers

**Figure 2:**
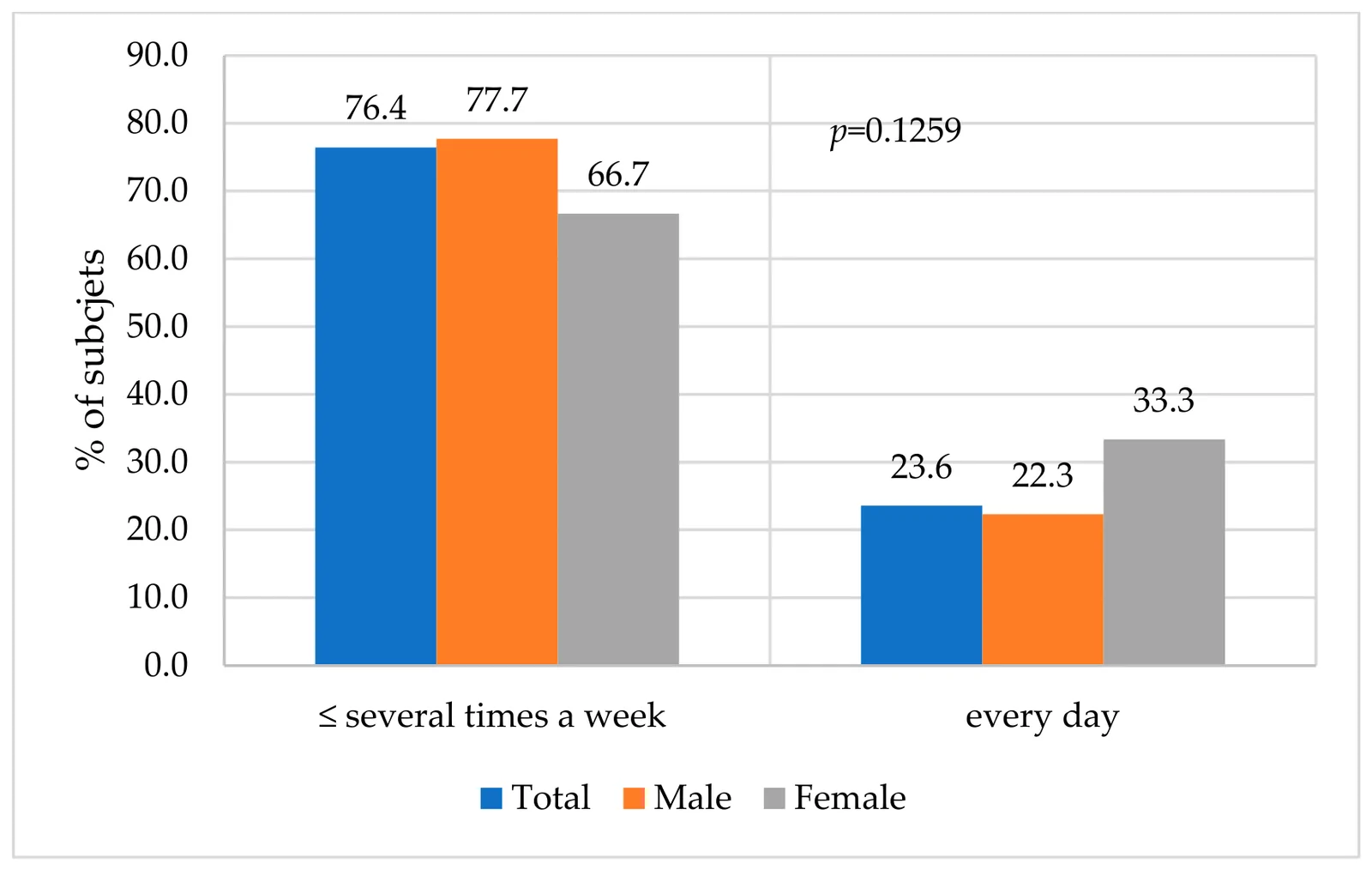
Frequency of vitamin D supplementation according to the soldiers’ sex.

The frequency of vitamin D supplementation and its association with 25(OH)D levels are shown in Figure 2 and Table 5. Daily vitamin D supplementation was reported by 23.6% of the respondents, without significant differences between men and women (*p* > 0.05). Vitamin D supplements were not provided to military personnel as part of a standardized supplementation program; supplementation was self-reported and reflected individual use of commercially available preparations. The exact dose and formulation of vitamin D supplements were not recorded in the questionnaire. Overall, 81 participants declared no vitamin D supplementation during the assessed period. For context, according to Polish recommendations, the commonly recommended vitamin D supplementation dose for adults is 1000–2000 IU/day, depending on body weight, dietary intake and sun exposure [5]. A significant correlation was demonstrated between the frequency of vitamin D supplementation and serum 25(OH)D concentration (*p* < 0.0001). Individuals supplementing vitamin D daily were characterized by a significantly higher concentration of 25(OH)D compared to those supplementing no more than several times a week (37.82 ± 13.64 vs. 30.82 ± 12.57 ng/mL; *p* < 0.0001).

**Figure 3:**
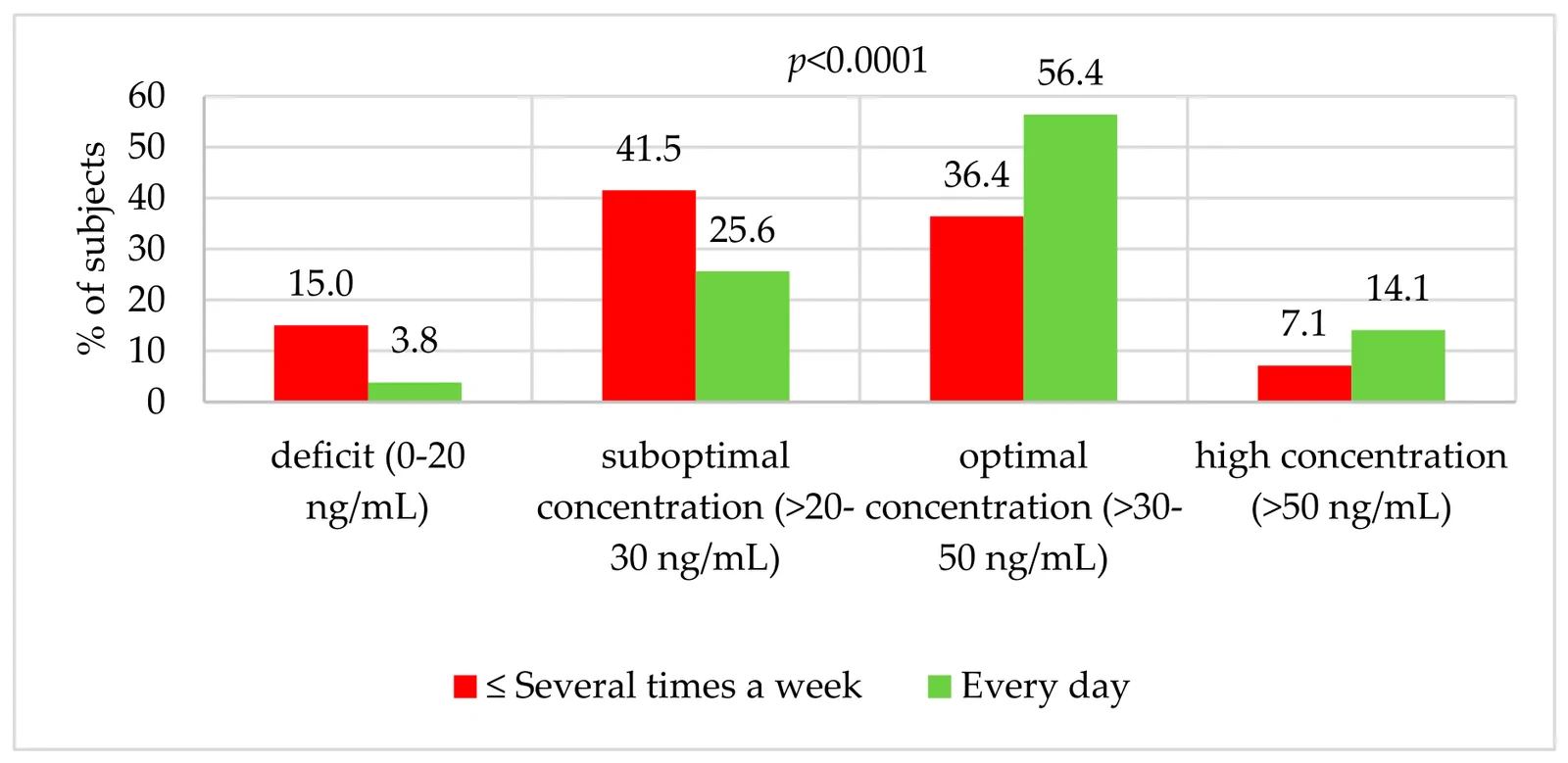
Relationship between vitamin D supplementation and vitamin D concentration in soldiers.

Soldiers declaring daily vitamin D supplementation were more likely to achieve sufficient concentrations of 25(OH)D according to the adopted classification (30–50 ng/mL) than those supplementing vitamin D no more than several times a week (56.4% vs. 36.4%) (Figure 3). In contrast, vitamin D deficiency (<20 ng/mL) occurred much less frequently in the daily supplementation group (3.8% vs. 15.0%), as did suboptimal concentrations (20 to <30 ng/mL) (25.6% vs. 41.5%). The percentage of subjects with 25(OH)D levels > 50 ng/mL was higher in the daily supplementation group than in those who supplemented less frequently (14.1% vs. 7.1%).

The season-stratified analysis showed significantly lower concentrations of 25(OH)D in subjects whose blood was collected for testing in the spring, compared to those tested in the summer and autumn period (30.99 ± 12.47 vs. 33.70 ± 13.00 ng/mL; *p* = 0.0293).

Due to the very small size of the study group in winter (*n* = 9), the group was not included in the seasonal analysis, which limits the possibility of fully assessing winter-related drops in 25(OH)D concentrations.

The frequency of vitamin D supplementation did not differ significantly between the sexes (χ^2^ test, *p* = 0.1259).

### 3.4. Associations of 25(OH)D with Mineral and Metabolic Parameters

Spearman’s correlation analysis (Table 6) was used to assess the associations between the concentration of 25(OH)D and the parameters of mineral and metabolic status. There was a significant negative correlation between the concentration of 25(OH)D and PTH (rho = −0.269; *p* < 0.001) and between 25(OH)D and homocysteine (rho = −0.134; *p* = 0.015). A weak positive correlation was also demonstrated between the concentration of 25(OH)D and inorganic phosphorus (rho = 0.109; *p* < 0.05). No significant correlation occurred between the concentration of 25(OH)D and total calcium.

### 3.5. Associations Between the GC rs2282679 Polymorphism and Serum 25(OH)D Concentrations

The following distribution of *GC* rs2282679 polymorphism genotypes was noted: TT—158 people (47.7%), TG—128 people (38.7%) and GG—45 people (13.6%). The frequency of the T allele was 0.671, while the G allele frequency was 0.329. The respective expected genotype frequencies, assuming the Hardy–Weinberg equilibrium, were: TT—148.9, TG—146.2 and GG—35.9. The distribution of genotypes was not consistent with the Hardy–Weinberg equilibrium (χ^2^ = 5.14; *df* = 1; *p* = 0.023), mainly due to the number of TG heterozygotes which was lower than expected and a higher number of TT and GG homozygotes.

The recessive genetic model (GG vs. TT + TG) was used as the primary genetic model because it corresponded to the subsequent multivariable logistic regression and classification tree analyses. Therefore, Table 7 presents the main genetic comparison, whereas Table 8 provides a complementary comparison across the three genotype groups (TT, TG and GG). The analysis of the recessive model showed a significantly lower concentration of 25(OH)D in GG homozygotes compared to subjects with TT and TG genotypes analyzed jointly (*p* = 0.0001; Table 7). In the comparative analysis of the three genotypic groups, 25(OH)D concentrations differed significantly between the TT, TG and GG groups (*p* = 0.0002; Table 8), with the lowest values observed in individuals with the GG genotype.

**Figure 4:**
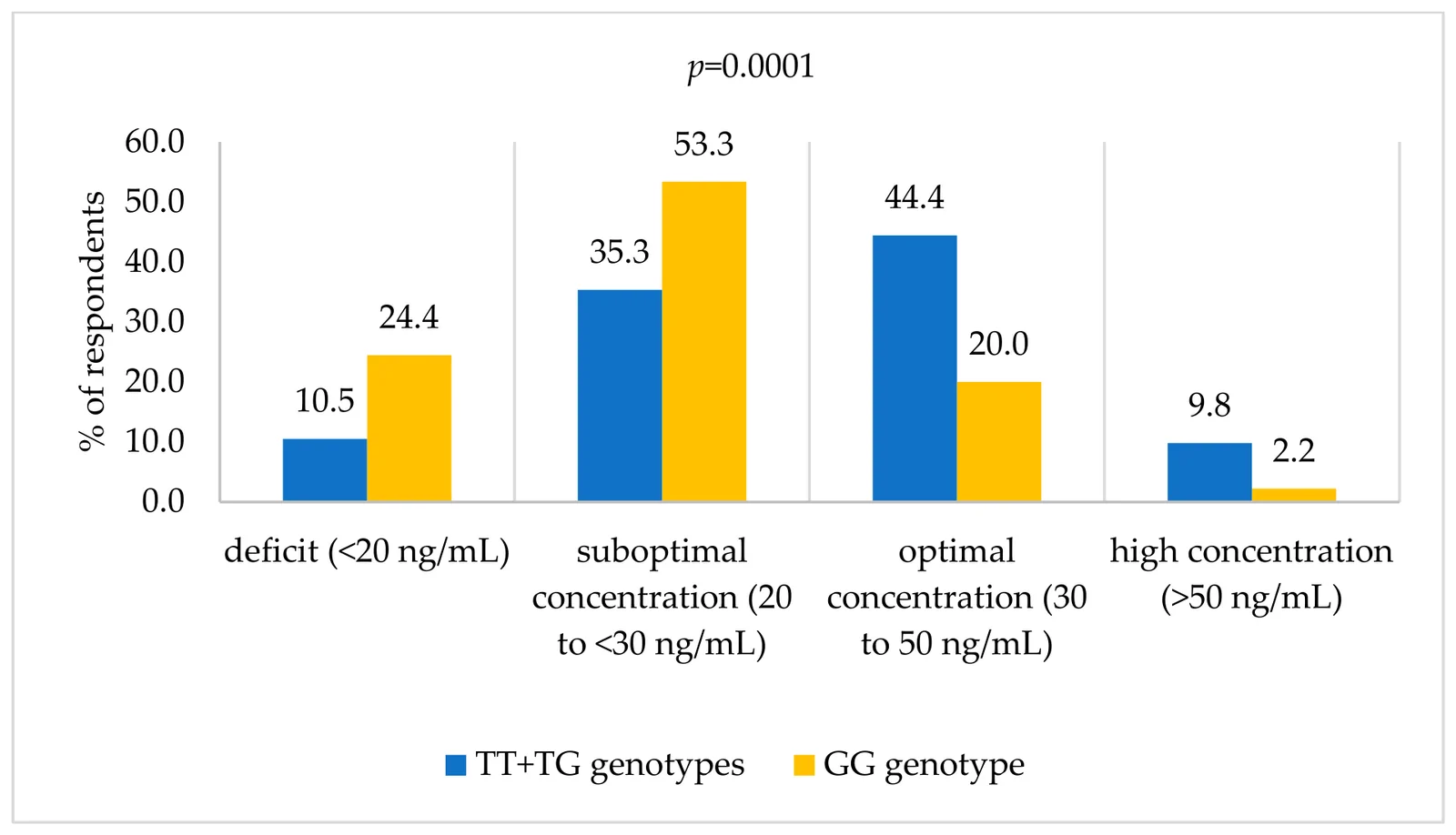
Distribution of serum 25(OH)D concentration categories according to the primary recessive model of GC rs2282679 (GG vs. TT + TG).

Consistently with this finding, vitamin D deficiency and suboptimal 25(OH)D concentrations were more frequently observed, whereas sufficient concentrations according to the adopted classification were less frequently observed in subjects with the GG genotype compared to those with the TT and TG genotypes analyzed jointly (*p* = 0.0001; Figure 4). Figure 4 presents vitamin D status categories according to the primary recessive genetic model (GG vs. TT + TG), which was also used in the subsequent multivariable logistic regression and classification tree analyses. The three-genotype comparison is presented separately in Table 8.

### 3.6. Factors Associated with Suboptimal Vitamin D Status—Multivariable Logistic Regression Analysis

Multivariable logistic regression was used to evaluate factors associated with suboptimal vitamin D status, defined as 25(OH)D concentrations < 30 ng/mL. The model included the age, sex, BMI, season when testing was performed, frequency of vitamin D supplementation and the genotype of the GC rs2282679 polymorphism analyzed in the recessive model (GG vs. TT + TG) (Table 9).

The multivariable analysis showed that performing tests in the spring was significantly associated with a higher risk of 25(OH)D concentration being <30 ng/mL compared to the summer and autumn period (OR = 1.99; 95% CI: 1.18–3.38; *p* = 0.010).

The frequency of vitamin D supplementation was also significantly associated with the risk of suboptimal vitamin D status. The risk of 25(OH)D < 30 ng/mL was over four times higher in participants declaring supplementation several times a week or less frequently compared to those supplementing daily (OR = 4.48; 95% CI: 2.37–8.47; *p* < 0.001).

The GG genotype of the GC rs2282679 polymorphism was significantly associated with a greater risk of suboptimal vitamin D status. Individuals with the GG genotype had a more than sixfold higher risk of 25(OH)D < 30 ng/mL than subjects with the TT or TG genotype (OR = 6.57; 95% CI: 2.87–15.01; *p* < 0.001).

No significant association was demonstrated between the age, sex, or BMI and the risk of suboptimal vitamin D status in the multivariable model. The results indicated that, in the studied military population, the risk of 25(OH)D concentrations being <30 ng/mL was primarily associated with the blood collection season, the frequency of supplementation and the GG genotype of the GC rs2282679 polymorphism.

### 3.7. Identification of Subgroups Associated with the Risk of Suboptimal Vitamin D Status Using a Classification Tree

**Figure 5:**
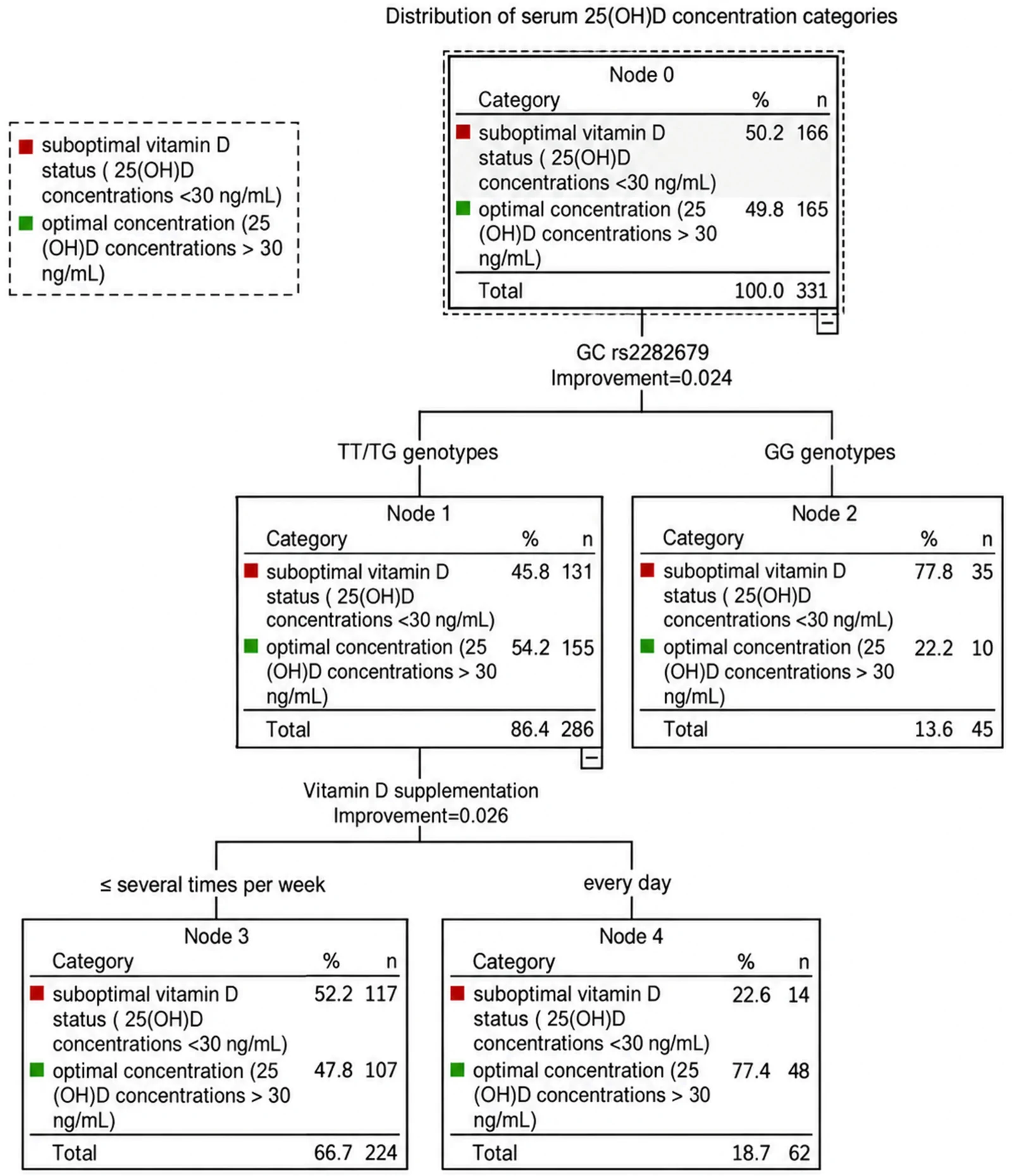
Classification tree identifying subgroups at different risk of suboptimal vitamin D status (serum 25(OH)D < 30 ng/mL).

An analysis using a classification tree was carried out (Figure 5) to identify subgroups differing in terms of the risk of suboptimal vitamin D status (defined as a concentration of 25(OH)D < 30 ng/mL). The model included the age, sex, BMI, season when testing was performed, frequency of vitamin D supplementation and the GC rs2282679 polymorphism analyzed in the recessive model (GG vs. TT + TG).

The genotype of the GC rs2282679 polymorphism was the first factor differentiating the respondents. As regards participants with the GG genotype, a concentration of 25(OH)D below 30 ng/mL was found in 77.8%, while in the group of people with the TT or TG genotypes, the percentage was 45.8%.

In the group with TT or TG genotypes, the frequency of vitamin D supplementation constituted another differentiating factor. Among those declaring daily supplementation, a concentration of 25(OH)D ≥ 30 ng/mL was observed in 77.4% of the respondents, while in the group supplementing vitamin D no more than several times a week, the percentage was 47.8%. The remaining variables included in the model, i.e., age, sex, BMI and the season when the blood was collected, were not selected as being contributory to subsequent divisions of the classification tree.

The structure of the tree allowed for distinguishing three subgroups for which 25(OH)D concentrations were compared (Table 10). The concentration of 25(OH)D differed significantly between the subgroups identified in the classification tree analysis (Kruskal–Wallis, *p* < 0.0001). The highest values were observed in individuals with TT/TG genotypes reporting daily vitamin D supplementation, intermediate in those with TT/TG genotypes supplementing vitamin D no more than several times a week, and the lowest in individuals with the GG genotype.

The obtained results indicated that the GG genotype of the *GC* rs2282679 polymorphism identified a subgroup with the least favorable concentration profile of 25(OH)D, while in individuals with TT or TG genotypes, the frequency of supplementation was an additional factor differentiating vitamin D status.

## 4. Discussion

The study aimed to assess the status of vitamin D in active Polish military personnel and to identify factors related to 25(OH)D concentration, including supplementation, diet, demographic and anthropometric factors, seasonality and the *GC* rs2282679 polymorphism. It was demonstrated that about half of the surveyed soldiers were deficient or suboptimal as regards vitamin D levels.

The independent factors associated with the suboptimal status of vitamin D were: blood collection in the spring, less frequent supplementation and the GG genotype of the *GC* rs2282679 polymorphism. No significant association was demonstrated between the age, sex, or BMI and the risk of suboptimal vitamin D status in the multivariable model. The frequency of the consumption of most analyzed sources of vitamin D was not significantly associated with 25(OH)D levels.

In the study population, the incidence of vitamin D deficiency was lower than in the general Polish population for which Płudowski et al. reported concentrations < 20 ng/mL in about 66% of the respondents [39]. The respective percentage in the present study was 12.4%. At the same time, suboptimal 25(OH)D concentrations, i.e., 20 to <30 ng/mL, were observed in 37.8% of the soldiers, indicating a persistent problem of suboptimal vitamin D status.

Similarly, a high incidence of vitamin D deficiency was also demonstrated in other military populations, including Portuguese soldiers and US military personnel [40]. Ruohola et al. [41] also showed that the concentration of 25(OH)D < 30.4 ng/mL was associated with over a threefold risk of stress fractures.

The above results highlighted the importance of adequate vitamin D status in the context of musculoskeletal health, limiting the risk of stress fractures and maintaining the physical fitness of military personnel [42,43,44,45]. According to Endocrine Society guidelines, maintaining 25(OH)D concentrations above 30 ng/mL in adults may require the supplementation of at least 1500–2000 IU of vitamin D per day [6,20].

In the US Army, attention was paid to the possibility of implementing nutritional interventions aimed at improving the bone health of recruits, an example of which is the Performance Readiness Bar—a snack fortified with calcium and vitamin D, used during basic military training [36]. A similar approach was described by Dyches et al., indicating that interventions involving calcium and vitamin D constituted a part of a strategy to reduce the risk of stress injuries in the military population [46].

The present study revealed no significant association between the consumption of most analyzed foods and the concentration of 25(OH)D. This result may confirm the limited role of diet in shaping vitamin D status compared to skin synthesis and supplementation. Despite being the main natural source of vitamin D, oily fish were eaten relatively rarely, which may have limited their effect on the concentration of 25(OH)D.

Low fish consumption is consistent with population data for Poland, remaining lower than in many European countries [47,48]. This may be due to both economic factors and established dietary habits.

A positive correlation was observed between egg intake and 25(OH)D concentration in men. Eggs are one of the few natural sources of vitamin D, containing both cholecalciferol and 25(OH)D. Similar observations were presented by Daly et al. [49], who indicated a potential impact of egg consumption on limiting seasonal decreases in 25(OH)D concentration.

In men, a weak positive correlation was confirmed between the use of omega-3 acid supplements and the concentration of 25(OH)D. This association may reflect the co-occurrence of health-promoting behaviors, including a greater propensity to take supplements on a regular basis, rather than the direct effect of omega-3 acids on vitamin D status. However, it cannot be ruled out that some supplements, especially those containing fish liver oil, may have served as an additional source of vitamin D. This result should be interpreted with caution and confirmed in analyses of the composition and doses of supplements used.

In women, a negative correlation was found between the frequency of the consumption of sweetened milk-based beverages and 25(OH)D concentrations. This result should be interpreted with caution due to the small size of the group of women and the exploratory nature of the correlation analysis. Sweetened milk products are not a typical or main source of vitamin D. Their composition may vary depending on the type of product and possible fortification [50,51]. In Poland, unlike margarines and fat spreads, milk and dairy products are not mandatorily fortified with vitamin D. Therefore, this association should be interpreted cautiously, as the FFQ did not include information on the fortification level of products. The negative trend in the association may reflect the co-occurrence of specific dietary or lifestyle patterns rather than the direct impact of this group of products on vitamin D status. The accidental nature of this observation cannot be ruled out either, especially in the context of the numerous nutritional variables analyzed. This association requires confirmation in larger research, taking account of the quantitative intake of vitamin D, information on product fortification and exposure to sunlight.

The frequency of supplementation was the strongest of the modifiable factors related to vitamin D status. Supplementing vitamin D several times a week or less frequently was associated with over a fourfold risk of 25(OH)D concentration < 30 ng/mL compared to daily supplementation. This result is consistent with the literature data indicating the key role of regular supplementation in maintaining sufficient 25(OH)D concentrations according to the adopted classification [5,18,52]. A meta-analysis of European clinical trials showed that oral vitamin D supplementation significantly increased the concentration of 25(OH)D in healthy adults, and the magnitude of the response depended on such factors as the dose and baseline concentration of 25(OH)D [53].

Seasonality was also important. Stratified analysis revealed that the concentration of 25(OH)D was lower in subjects whose blood was collected in the spring, compared to those whose blood was collected in the summer and autumn. In addition, based on the multivariable analysis, blood collection in the spring was associated with almost double the risk of 25(OH)D < 30 ng/mL. This observation corresponds to the phenomenon referred to as “post-winter depletion” resulting from limited skin synthesis in the winter and the gradual depletion of body reserves [54,55,56]. These results are consistent with population-based observations indicating seasonal fluctuations in 25(OH)D concentrations in Europe [50]. In the military population, this effect may be further intensified by limited exposure to UV radiation resulting from wearing uniforms and working indoors, despite high levels of physical activity. As all participants were based in Poland and deployed overseas during the study-related sampling period, the season of blood collection may be interpreted as a crude proxy for seasonal UVB availability in Poland. However, individual sunlight exposure, time spent outdoors, and sunscreen use were not recorded. Therefore, season should be considered an indirect indicator rather than a direct measure of sun exposure.

The present study demonstrated a significant association between the *GC* rs2282679 polymorphism and 25(OH)D concentration. In the recessive model, individuals with the GG genotype were characterized by a significantly lower concentration of 25(OH)D compared to those with TT and TG genotypes analyzed jointly. The comparative analysis of the three genotypic groups also showed significant differences in 25(OH)D concentrations among the TT, TG and GG groups, with the lowest values being observed in GG homozygotes. The results are consistent with previous reports indicating the participation of the rs2282679 variant in the regulation of vitamin D-binding protein concentration and 25(OH)D status [12,31,57,58].

The biological plausibility of this association may be explained by the role of DBP in vitamin D transport. DBP, encoded by the *GC* gene, is the main carrier protein for circulating vitamin D metabolites, including 25(OH)D. Therefore, genetic variations in the *GC* gene may influence total serum 25(OH)D concentrations by affecting DBP concentration, binding affinity, transport efficiency, or the stability and half-life of DBP-bound vitamin D metabolites [12,31,57,58]. The rs2282679 variant is an intronic marker and may not itself be the causal variant; however, it may tag functional *GC* haplotypes that influence DBP expression or DBP-related properties. In this context, lower 25(OH)D concentrations observed among GG homozygotes in both the recessive model and the three-genotype comparison are biologically plausible. Since DBP concentration, free 25(OH)D, and bioavailable 25(OH)D were not measured in the present study, this mechanism should be interpreted cautiously and confirmed in future studies.

Multivariable analysis confirmed the importance of this association. The GG genotype was associated with over a sixfold increase in the risk of 25(OH)D concentrations <30 ng/mL compared to TT and TG genotypes analyzed jointly. This result indicates that the homozygous GG genotype may identify a subgroup particularly susceptible to the risk of suboptimal vitamin D status.

The results of the classification tree analysis further indicate the hierarchical importance of genetic and behavioral factors in shaping vitamin D status. The *GC* rs2282679 genotype analyzed in the recessive model was the first differentiating factor in the study group. Individuals with the GG genotype formed a subgroup with the highest percentage of 25(OH)D concentrations < 30 ng/mL and the lowest concentration of 25(OH)D. In individuals with TT or TG genotypes, the frequency of vitamin D supplementation was another differentiating factor. The highest 25(OH)D concentrations were observed in individuals with TT/TG genotypes supplementing vitamin D on a daily basis, intermediate in those supplementing vitamin D less frequently, and the lowest in individuals with the GG genotype. These results suggest that the GG genotype identified a subgroup with a particularly unfavorable vitamin D status, whereas in individuals with TT or TG genotypes, regular supplementation constituted an additional differentiating factor. However, this observation requires confirmation in prospective studies, taking into account supplementation doses and formal analyses of genotype-supplementation interactions.

No significant associations occurred between the concentration of 25(OH)D and age and sex. This lack of an independent association between age and 25(OH)D status is consistent with recent findings by Borecka et al., who showed that vitamin D status in healthy older adults might be comparable to that in younger adults when differences in sunlight exposure, outdoor activity and dietary vitamin D intake are taken into account. Moreover, skin 7-dehydrocholesterol concentration and the serum vitamin D_3_ response to UVR may be similar in healthy older and younger adults [59,60]. Similar observations were presented by Câmara et al. [61], who did not confirm such a relationship either. In contrast to our results, Kelly et al. [62] reported higher rates of vitamin D deficiency in female military personnel and in older age groups. The association between age and vitamin D status was also observed in population studies conducted outside the military environment [14,63]. Minter et al. demonstrated a higher incidence of vitamin D deficiency in men than in women [64], as did Cheng et al. [65]. The lack of significant associations in the present study may result from the predominant role of supplementation, seasonality, and genetic factors. In the case of sex, we also cannot rule out limited statistical power resulting from the relatively low number of women in the study group.

As regards BMI, the obtained results partially correspond to previous research indicating a higher risk of suboptimal vitamin D status in individuals with excessive body weight [14,15,58,66]. The relationship between body weight and the concentration of 25(OH)D is complex and may result from such factors as the sequestration of vitamin D in the adipose tissue, its modified distribution and limited bioavailability [67,68]. According to current recommendations, obese individuals may require higher supplementation doses than those with normal body weight [5,69,70]. Moreover, previous analyses indicated that weight reduction might be associated with increased concentrations of 25(OH)D, which further confirms the multifactorial nature of the relationship between obesity and vitamin D status [71].

In this study, higher BMI categories and higher body fat content were associated with lower concentrations of 25(OH)D in univariate analyses. However, BMI did not remain a significant factor associated with the risk of suboptimal vitamin D status in the multivariable model. Therefore, the importance of BMI might be weaker in the study population than the importance of other factors, such as the blood collection season, the frequency of supplementation and the GG genotype of the GC rs2282679 polymorphism. At the same time, the lack of detailed data on supplement doses limits the possibility of assessing whether supplementation was adequate for the body weight of the study group soldiers.

Spearman’s correlation analysis showed a significant negative association between 25(OH)D and both PTH and homocysteine. The inverse association between 25(OH)D and PTH is consistent with the physiology of the vitamin D–parathyroid axis. Homocysteine was included in the analysis as an available metabolic marker because elevated homocysteine concentrations are associated with increased cardiovascular and neurological risk, and previous studies suggested an inverse relationship between homocysteine and vitamin D status [25,72,73,74,75]. In the present study, the negative correlation between 25(OH)D and homocysteine was weak but statistically significant, which is consistent with those observations. However, due to the cross-sectional design of the study, this association should not be interpreted causally. Moreover, the present study was not designed as a comprehensive cardiovascular risk assessment, nor were other cardiovascular risk factors analyzed in the population. Therefore, the observed association between 25(OH)D and homocysteine should be interpreted as exploratory.

A weak positive correlation was also found between 25(OH)D and inorganic phosphorus, which may reflect the contribution of vitamin D to the regulation of phosphate metabolism. However, due to the low strength of this correlation, the result should also be interpreted with caution.

The lack of a significant correlation between the concentration of 25(OH)D and total calcium may result from compensatory mechanisms that maintain calcium homeostasis.

The obtained results indicate that the status of vitamin D in the military population is shaped by the interaction of environmental, behavioral and genetic factors. The most significant factors in the study population were the frequency of supplementation, seasonality and the GG genotype of the GC rs2282679 polymorphism. The results of the classification tree analysis additionally indicate that the factors may act hierarchically, and the GG genotype may identify a group that is particularly susceptible to unfavorable vitamin D status.

From the perspective of military medicine, maintaining proper vitamin D status may be important to reduce the risk of injury, support musculoskeletal health, and maintain the physical fitness and operational readiness of military personnel.

## 5. Strengths and Limitations

The most important strengths of this study include a comprehensive assessment of factors related to vitamin D status in the military population. Unlike many previous studies analyzing single determinants of 25(OH)D concentration, this paper simultaneously comprised nutritional factors, supplementation, seasonality, anthropometric and demographic characteristics, and genetic conditions. Such an approach enabled a multidimensional assessment of factors associated with suboptimal vitamin D status. An important advantage of the study is the analysis of active military personnel, which is a relatively rarely studied population in the context of vitamin D status, especially in Europe. The obtained results may be of practical significance for preventive actions aimed at maintaining the health, physical fitness and operational readiness of soldiers.

An additional strength of the study was the inclusion of the analysis of the GC rs2282679 polymorphism encoding vitamin D-binding protein (DBP), which made it possible to assess the association between genetic predisposition and the risk of suboptimal vitamin D status. Furthermore, the use of multivariable logistic regression allowed for the identification of factors independently related to the concentration of 25(OH)D < 30 ng/mL after accounting for potential confounding factors.

However, the presented results should be interpreted taking into account several limitations. Firstly, genetic analysis included only the rs2282679 polymorphism of the *GC* gene. Although this variant is one of the most thoroughly described markers associated with 25(OH)D concentrations, vitamin D metabolism is polygenic and encompasses numerous genes involved in its synthesis, transport, activation, and degradation. Therefore, the influence of other genetic variants cannot be ruled out, including those occurring in the *VDR*, *CYP2R1*, *CYP27B1,* and *CYP24A1* genes.

Secondly, the distribution of *GC* rs2282679 polymorphism genotypes deviated from the Hardy–Weinberg equilibrium. This deviation may result from the specific structure of the study population, including active military personnel, which does not constitute a random sample of the general population, as well as from potential selection effects related to the health and fitness requirements of military personnel. However, the influence of population structure or technical limitations of genetic analysis may not be entirely excluded. Therefore, the observed association between rs2282679 and vitamin D status should be interpreted with caution and confirmed in larger studies.

Thirdly, the Food Frequency Questionnaire (FFQ) was used to evaluate the diet. This method makes it possible to assess long-term eating habits. However, it is based on self-reported data and is subject to the risk of memory errors and reporting inaccuracies. In addition, the FFQ does not allow for the precise estimation of the actual intake of vitamin D or the size of the consumed portions.

The lack of detailed data on the doses of vitamin D supplements used is another limitation. Only the frequency of supplementation was considered in the analysis, which makes it impossible to assess the dose–response relationship between supplementation and the concentration of 25(OH)D.

The cross-sectional nature of the study also constitutes a limitation, as it does not allow causal relationships between the analyzed factors and vitamin D status to be inferred. In addition, other cardiovascular risk factors were not analyzed; therefore, the association between 25(OH)D and homocysteine should be interpreted as exploratory and requires confirmation in studies designed specifically to assess cardiovascular risk.

Moreover, the size of the group of people whose blood was collected in the winter was very small (n = 9). Therefore, this group was not included in the seasonal analysis. This limits the possibility of fully evaluating winter-related declines in 25(OH)D concentration and year-round variability in vitamin D status. This limitation resulted from the implementation schedule of the program, under which the recruitment and collection of biological material was carried out.

Individual exposure to sunlight, the use of photoprotective agents, and time spent outdoors, which may considerably affect skin vitamin D synthesis and constitute potential uncontrolled confounding factors, have not been evaluated either. Skin phototype was not formally assessed; however, all participants were Polish soldiers of European origin.

The study population included only 11.8% women, which could limit the possibility of detecting sex-specific associations and reduce the possibility of generalizing the results to the population of women performing military service. Nevertheless, the gender structure reflects the actual participation of women in the Polish Armed Forces, which increases the representativeness of the sample for the professional population of soldiers.

Finally, the study population had a specific nature (military personnel), which may limit the application of the results to the general population.

## 6. Conclusions

1. Approximately half of the study group soldiers were found to be deficient or suboptimal in vitamin D, which indicates the common occurrence of suboptimal vitamin D status in this population. Vitamin D status was primarily associated with seasonality, frequency of supplementation, and the *GC* rs2282679 polymorphism.
2. BMI and body fat content were associated with 25(OH)D concentrations in univariate analyses. However, they were not independent factors associated with the risk of suboptimal vitamin D status in the multivariable model. No independent associations were demonstrated between age or sex and the risk of suboptimal vitamin D status. The frequency of the consumption of most analyzed sources of vitamin D was not significantly associated with 25(OH)D levels.
3. 25(OH)D concentrations were negatively associated with parathyroid hormone and homocysteine concentrations and weakly positively associated with inorganic phosphorus concentrations. No significant association was noted between the concentration of 25(OH)D and total calcium.
4. The GG genotype of the *GC* rs2282679 polymorphism was associated with a 6.5-fold increased risk of 25(OH)D concentrations being <30 ng/mL compared to TT and TG genotypes.
5. The analysis of the classification tree indicated that the GG genotype of the *GC* rs2282679 polymorphism identified the subgroup with the lowest 25(OH)D concentration and the highest incidence of <30 ng/mL values. As regards individuals with TT or TG genotypes, daily vitamin D supplementation was associated with the highest 25(OH)D concentrations.
6. The obtained results indicate that the analysis of the *GC* rs2282679 polymorphism combined with information on vitamin D supplementation and seasonality may support the identification of individuals particularly exposed to suboptimal vitamin D status. However, further prospective studies in the field of nutrigenetics are needed to assess the clinical usefulness of information on this genetic variant in the individualization of recommendations for prevention and vitamin D supplementation.
7. Based on the obtained results, we recommend considering the determination of 25(OH)D concentrations in recruits starting military service and as part of periodic medical assessment of soldiers. Currently, serum 25(OH)D determination is not performed as part of standard diagnostics at any stage of military service.

## Figures and Tables

**Table 1:** Demographic and anthropometric characteristics of the study population.

Variables	Total (N = 331)					Male (N = 292)	Female (N = 39)	
	M	SD	Me (IQR)	Min	Max	Me (IQR)	Me (IQR)	U Mann–Whitney Test *p*-Value
Age [years]	39.4	9.3	39.0 (32.0–46.0)	21.0	65.0	40.0 (32.0–47.0)	36.0 (32.0–40.0)	0.0371
Height [cm]	178.5	7.9	179.0 (173.0–183.5)	153.0	200.0	180.0 (176.0–184.0)	167.0 (163.0–169.0)	<0.0001
Weight [kg]	88.0	17.1	87.4 (78.0–96.9)	49.7	182.1	89.5 (80.3–97.8)	63.7 (57.1–70.8)	<0.0001
BMI [kg/m^2^]	27.5	4.7	26.8 (24.4–29.9)	18.1	53.2	27.1 (24.8–30.1)	22.3 (20.6–26.3)	<0.0001
BF [%]	22.5	6.3	21.7 (18.0–26.4)	9.9	46.1	21.1 (17.2–25.6)	25.9 (23.6–33.1)	<0.0001
BF [kg]	20.4	9.3	18.3 (14.2–24.2)	6.7	83.9	18.7 (14.2–24.3)	16.3 (13.4–23.8)	0.2807
MM [kg]	64.2	10.0	66.2 (59.6–70.2)	37.0	93.4	66.8 (61.5–70.8)	44.8 (41.2–48.2)	<0.0001
LBM [kg]	67.6	10.4	69.6 (62.8–73.8)	39.0	98.2	70.3 (64.9–74.5)	47.2 (43.4–50.8)	<0.0001
TBW [kg]	48.2	7.6	49.1 (44.6–52.8)	27.8	74.1	49.7 (46.2–53.1)	33.8 (31.0–36.3)	<0.0001
EW [kg]	19.5	2.8	19.7 (18.2–21.1)	11.8	31.7	20.1 (18.7–21.3)	14.3 (13.1–15.6)	<0.0001
IW [kg]	28.7	4.9	29.3 (26.2–31.7)	15.7	43.6	29.6 (27.5–32.1)	19.5 (17.8–21.2)	<0.0001
WC [cm]	95.5	13.7	94.0 (86.0–105)	65.0	158.0	95.5 (88.0–105.0)	78.0 (71.0–85.0)	<0.0001
HC [cm]	105.1	9.1	104.0 (100–109)	90	197.0	104.0 (100.0–109.0)	99.0 (95.0–107.0)	0.0002
25(OH)D [ng/mL]	32.5	13.1	29.9 (24.0–38.1)	11.4	90.2	29.7 (23.9–37.4)	32.9 (25.0–43.0)	0.1076
Total calcium [mg/dL]	9.7	0.5	9.8 (9.5–10.0)	8.9	11.3	9.8 (9.5–10.0)	9.7 (9.5–9.9)	0.3430
Phosphorus [mmol/L]	1.0	0.3	1.0 (0.9–1.1)	0.6	3.9	1.0 (0.9–1.1)	1.1 (1.0–1.2)	0.0170
PTH [pg/mL]	32.7	11.0	31.3 (25.54–38.13)	13.0	77.4	31.3 25.6–38.2	32.1 (24.9–36.7)	0.7811
HCY [µmol/L]	12.3	3.4	12.1 (10.2–14.3)	0.9	27.5	12.2 (10.2–14.5)	11.6 (10.1–14.1)	0.2230

Abbreviations: N—group size, BMI—Body Mass Index, BF—Body Fat, MM—Muscle Mass, LBM—Lean Body Mass, TBW—Total Body Water, EW—Extracellular Water, IW—Intracellular water, WC—Waist Circumference, HC—Hip Circumference, PTH—Parathyroid hormone, HCY—Homocysteine, M—Mean, SD—Standard Deviation, Me—Median, IQR—Interquartile Range, *p* < 0.05 level.

**Table 2:** Prevalence of overweight and obesity according to BMI and body fat percentage in the study group of soldiers.

Variable	Category	Total (N = 331)		Male (N = 292)		Female (N = 39)		
		N	%	N	%	N	%	Chi^2^ *p*-Value
BMI category	Normal 18.5 to <25	104	31.4	78	26.7	26	66.7	<0.0001
	Overweight 25 to <30	148	44.7	140	47.9	8	20.5	
	Obese ≥ 30	79	23.9	74	25.3	5	12.8	
Body fat category	Underfat	6	1.8	0	0	6	15.4	<0.0001
	Normal	147	44.4	124	42.5	23	59.0	
	Overfat	99	29.9	89	30.5	10	25.6	
	Obese	79	23.9	79	27.1	0	0	

Abbreviations: N—group size, *p* < 0.05 level.

**Table 3:** Total Vitamin D concentration in the soldiers’ blood in relation to BMI and body fat.

Group	Category	N	Me (IQR)	M ± SD	Min–Max	ANOVA Kruskal–Wallis *p*-Value
BMI	Normal weight	104	31.53 (24.42–41.04)	34.38 ± 14.25	13.00–90.19	0.0051
	Overweight	148	29.97 (25.00–38.20)	33.30 ± 13.28	11.40–82.79	
	Obese	79	27.32 (20.00–34.00)	28.39 ± 10.40	11.55–66.00	
Body fat	Underfat/Normal	153	31.96 (25.00–41.93)	34.54 ± 13.96	13.00–90.19	0.004
	Overweight	99	29.35 (24.04–35.00)	32.47 ± 13.08	12.55–82.79	
	Obese	79	27.52 (20.00–34.86)	28.45 ± 10.60	11.40–67.33	

Abbreviations: N—group size, M—mean, SD—standard deviation, Me—median, IQR—Interquartile Range, Min—minimum, Max—maximum, *p* < 0.05 level.

**Table 4:** Frequency of the consumption of selected food products that are dietary sources of vitamin D in the group of soldiers.

In the Past 12 Months, How Often Did You Drink or Eat:	Never or Almost Never	Once a Quarter or Less Frequently	Once a Month or Less	Several Times a Month	Once a Week	Several Times a Week	Every Day	Several Times a Day
Oily fish, e.g., salmon, sardines, herring, mackerel, large carp, eel	3.0%	14.3%	27.1%	32.8%	17.9%	4.6%	0.3%	0.0%
Lean fish, e.g., pollock, cod, perch, hake, carp, tuna, panga, trout	2.1%	15.2%	32.2%	28.3%	17.9%	4.0%	0.3%	0.0%
Eggs	0.0%	0.0%	2.1%	9.7%	22.2%	53.5%	11.2%	1.2%
Oil (all kinds)	0.6%	3.0%	8.2%	17.4%	12.5%	45.1%	12.5%	0.6%
Butter (all types)	6.8%	6.5%	8.7%	9.0%	7.1%	34.2%	25.8%	1.9%
Margarine in cubes (for baking, frying), margarine in cups (for spreading)—all types	47.1%	16.7%	11.2%	7.9%	3.6%	8.2%	4.9%	0.3%
Cream (acidified or sweet) for dishes or drinks	11.6%	10.6%	18.2%	31.3%	11.6%	14.9%	1.8%	0.0%
Other animal fats such as lard	29.6%	23.2%	20.4%	15.2%	7.6%	3.7%	0.3%	0.0%
Mayonnaise and dressings, i.e., salad dressings—all types	5.2%	16.8%	20.1%	33.2%	11.0%	12.5%	1.2%	0.0%
Milk and natural dairy beverages, e.g., milk, milk soups, natural yogurt, kefir, natural buttermilk	1.5%	2.1%	4.6%	13.1%	11.9%	34.7%	26.1%	6.1%
Sweetened dairy beverages, e.g., fruit yogurts, yogurts with chocolate flakes, flavored buttermilk, fruit yogurt, cocoa with milk	11.6%	10.7%	17.4%	21.6%	12.8%	21.0%	4.0%	0.9%
Cheeses, e.g., yellow cheese, blue cheeses, melted cheeses, cheese spreads	1.8%	4.0%	5.8%	17.4%	14.3%	44.5%	10.1%	2.1%
Natural cottage cheeses, e.g., assorted cottage cheeses, natural cottage cheese, mozzarella, cottage cheese with herbs	1.5%	2.1%	7.0%	23.7%	17.9%	37.4%	9.4%	0.9%
Flavored cottage cheeses, e.g., fruit, chocolate, vanilla	30.7%	14.9%	22.2%	16.1%	7.0%	7.6%	1.5%	0.0%
Omega-3 fatty acids	38.2%	11.9%	9.5%	11.3%	4.6%	9.8%	14.7%	0.0%

**Table 5:** Serum 25(OH)D concentrations according to vitamin D supplementation frequency and the season of blood sampling.

Variable	Group	N	Me (IQR)	M ± SD	Min–Max	U Mann–Whitney Test *p*-Value
Supplementation	≤several times a week	253	29.00 23.00–34.95	30.82 ± 12.57	11.4–90.19	<0.0001
	Every day	78	36.0 29.00–44.70	37.82 ± 13.64	14.00–89.00	
Season	Spring	99	29.00 21–38	30.99 ± 12.47	11.4–89.00	0.0293
	Summer/Autumn	223	31.00 25.00–39.04	33.70 ± 13.00	15.00–90.19	

Abbreviations: N—group size, M—mean, SD—standard deviation, Me—median, IQR—Interquartile Range, Min—minimum, Max—maximum, *p* < 0.05 level.

**Table 6:** Correlations between 25(OH)D concentration and mineral and metabolic status parameters.

Variable	25(OH)D	
	Rho	*p*
Calcium	0.039	0.476
Phosphorus	0.109	0.047
Parathyroid hormone	−0.269	<0.001
Homocysteine	−0.134	0.015

Spearman’s rank correlation coefficient was used. Statistical significance was set at *p* < 0.05.

**Table 7:** Association of GC rs2282679 with serum 25(OH)D under the primary recessive model (GG vs. TT + TG).

Genotype Group	N	Me (IQR)	25(OH)D [ng/mL] M ± SD	Min–Max	U Mann–Whitney Test *p*-Value
TT + TG	286	31.00 (25.0–39.04)	33.38 ± 13.39	11.40–90.19	0.0001
GG	45	24.00 (20.07–29.23)	26.65 ± 9.77	16.0–62.70	

Abbreviations: N—group size, M—mean, SD—standard deviation, Me—median, IQR—Interquartile Range, Min—minimum, Max—maximum, *p* < 0.05 level.

**Table 8:** Serum 25(OH)D concentrations across GC rs2282679 genotype groups.

Genotype Group	N	Me (IQR)	25(OH)D [ng/mL] M ± SD	Min–Max	ANOVA Kruskal–Wallis *p*-Value
TT	158	31.06 (25.70–41.75)	34.09 ± 13.71	11.55–90.19	0.0002
TG	128	30.00 (24.00–38.00)	32.51 ± 12.98	11.40–89.00	
GG	45	24.00 (20.07–29.23)	26.65 ± 9.77	16.00–62.70	

Abbreviations: N—group size, M—mean, SD—standard deviation, Me—median, IQR—Interquartile Range, Min—minimum, Max—maximum, *p* < 0.05 level.

**Table 9:** Multivariable logistic regression model for serum 25(OH)D concentration < 30 ng/mL in the studied soldiers.

Variable	B	SE	*p*	OR	95% CI
Age (years)	0.007	0.014	0.614	1.01	0.98–1.03
Sex (female vs. male)	0.391	0.408	0.338	1.48	0.66–3.29
Season (spring vs. summer/autumn)	0.690	0.269	0.010	1.99	1.18–3.38
Vitamin D supplementation (≤several times per week vs. every day)	1.500	0.325	<0.001	4.48	2.37–8.47
GC rs2282679 (GG vs. TT + TG)	1.882	0.422	<0.001	6.57	2.87–15.01
BMI (kg/m^2^)	−0.027	0.030	0.355	0.97	0.92–1.03

Abbreviations: B—regression coefficient; SE—standard error; OR—odds ratio; CI—confidence interval; *p* < 0.05 level.

**Table 10:** Vitamin D Total concentration in the soldiers’ blood according to subgroups identified with classification trees.

Classification Tree Subgroups	N	Me (IQR)	25(OH)D [ng/mL] M ± SD	Min–Max	ANOVA Kruskal–Wallis *p*-Value
TT/TG genotypes, daily vitamin D supplementation	62	37.50 (30.00–45.13)	39.65 ± 13.85	14.00–89.00	<0.0001
TT/TG genotypes, Vitamin D supplementation ≤ several times per week	224	29.72 (23.95–36.12)	31.65 ± 12.75	11.40–90.19	
GG genotypes	45	24.00 (20.07–29.23)	26.65 ± 9.77	16.00–62.70	

Abbreviations: N—group size, M—mean, SD—standard deviation, Me—median, IQR—Interquartile Range, Min—minimum, Max—maximum, *p* < 0.05 level.

## Data Availability

The data are not publicly available because they concern active-duty military personnel and are subject to institutional and confidentiality restrictions. De-identified data may be made available from the corresponding author upon reasonable request and subject to approval by the relevant institutional authorities.

## Supplementary Materials

The following supporting information can be downloaded at: https://www.mdpi.com/article/10.3390/nu18183041/s1. Table S1: Spearman’s correlations between the frequency of consumption of selected vitamin D-source foods and serum 25(OH)D concentrations by sex.
