# Supplementary material for: Vitamin D Status in Polish Military Personnel: Associations with Supplementation, Seasonality, and the GC rs2282679 Polymorphism
Source: Nutrients. 2026 Sep 17;18(18):3041. doi: 10.3390/nu18183041 (PMC13610348; doi:10.3390/nu18183041)
Supplement: Supplementary file 1 [file nutrients-18-03041-s001.zip › nutrients-4526387-supplementary.pdf]

SUPPLEMENTARY

Table S1. Spearman's correlations between the frequency of the consumption of selected foods that are sources of vitamin D and vitamin D concentrations in soldiers by sex.

**Selected foods that are sources of vitamin D**	**Total (n=331)**		**Men (n=292)**		**Women (n=39)**	
	R	p	r	p	r	p
Lean fish, e.g., pollock, cod, perch, hake, carp up to 1 kg, tuna, panga, trout	0.073	0.186	0.104	0.076	-0.135	0.413
Oily fish, e.g., salmon, sardines, herring, mackerel, large carp, eel	0.051	0.352	0.057	0.336	-0.011	0.949
Oil (all types)	0.112	0.042	0.105	0.074	0.145	0.380
Butter (all types)	0.052	0.348	0.075	0.208	-0.159	0.334
Margarine in cubes (for baking, frying), margarine in cups (for spreading) – all types	-0.082	0.137	-0.060	0.307	-0.226	0.167
Cream (acidified or sweet) for dishes or drinks	0.056	0.310	0.050	0.392	0.100	0.545
Other animal fats such as lard	0.015	0.787	0.008	0.897	0.131	0.427
Mayonnaise and dressings, i.e., salad dressings – all types	0.032	0.564	0.012	0.845	0.236	0.149
Milk and natural dairy drinks, e.g., milk soups, natural yogurt, kefir, natural buttermilk	0.078	0.157	0.089	0.129	-0.013	0.936
Sweetened dairy beverages, e.g., fruit yogurts, chocolate flake yogurts, flavored buttermilk, fruit yogurts	-0.071	0.203	-0.032	0.584	-0.381	0.017
Natural cottage cheeses, e.g., various cottage cheeses, natural cottage cheese, mozzarella, cottage cheese with herbs	0.084	0.126	0.087	0.141	0.021	0.898
Flavored cottage cheese, e.g., fruit, chocolate, vanilla	-0.102	0.066	-0.076	0.194	-0.231	0.156
Cheeses, e.g., yellow cheeses, mould cheeses, melted cheeses, cheese spreads	-0.035	0.527	-0.053	0.367	0.096	0.559
Eggs and egg dishes, in which eggs are the primary ingredient of the dish, e.g., scrambled eggs, omelet, egg paste	0.163	0.003	0.167	0.004	0.182	0.266
Omega-3 fatty acids	0.141	0.011	0.149	0.011	0.240	0.141
